# Pharmacokinetic Profile of Extracts from the Chayote (*Sechium edule*) H387 07 Hybrid and Phytochemical Characterization of Its Segregant H387 M16 for Potential Therapeutic Applications

**DOI:** 10.3390/molecules30193948

**Published:** 2025-10-01

**Authors:** Eugenia Elisa Delgado-Tiburcio, Ramón Marcos Soto-Hernández, Itzen Aguiñiga-Sánchez, Jorge Cadena-Iñiguez, Lucero del Mar Ruiz-Posadas, Cecilia B. Peña-Valdivia, Héctor Gómez-Yáñez

**Affiliations:** 1 Botany Department, Postgraduate College Campus Montecillo, km 36.5 Carretera México-Texcoco, Texcoco 56230, Mexico; delgado.eugenia@colpos.mx (E.E.D.-T.); cecilia@colpos.mx (C.B.P.-V.); gomez.hector@colpos.mx (H.G.-Y.); 2 Hematopoiesis and Leukemia Laboratory, Research Unit on Cell Differentiation and Cancer, FES Zaragoza, National Autonomous University of Mexico, Mexico City 09230, Mexico; liberitzen@comunidad.unam.mx; 3 Innovation in Natural Resource Management, Postgraduate College, Campus San Luis Potosí, Salinas de Hidalgo, San Luis Potosí 78622, Mexico

**Keywords:** pharmacokinetics, *Sechium edule*, chayote, hybrid, secondary metabolites, cucurbitacins, flavonoids, antioxidant activity, anti-inflammatory activity, anticancer potential

## Abstract

The hybrid *Sechium edule* H387 07, commonly known as chayote, has shown potential as an antiproliferative, cytotoxic, and pro-apoptotic agent in the murine leukemia cell lines P388 (macrophagic) and J774 (monocytic) and in the myelomonocytic leukemia cell line WEHI-3. However, despite these reported bioactivities, its pharmacokinetic profile remains largely unexplored. Understanding the absorption, distribution, and elimination of this hybrid is critical for addressing unmet therapeutic needs and for advancing the development of natural product-based therapies. These effects are attributed to the presence of phenols, flavonoids, and cucurbitacins in its organic extracts. In this study, the pharmacokinetic parameters of secondary metabolites from methanolic extracts of *Sechium* H387 07 were evaluated after oral administration in mice, while its segregant H387 M16 was subjected to complementary phytochemical characterization. Methanolic extracts of *Sechium edule* H387 07 were orally administered to mice at doses of 8, 125, and 250 mg/kg, and plasma, liver, and urine samples were collected at 1, 6, 24, and 48 h post-treatment. High-performance liquid chromatography (HPLC) identified polyphenols and cucurbitacins, notably cucurbitacin B (CuB) and cucurbitacin IIA (CuIIA), in the biological samples, and pharmacokinetic variables such as the maximum plasma concentration (C_max_), time to reach maximum concentration (T_max_), half-life (T_1/2_), and volume of distribution (V_d_) were determined. For instance, CuB exhibited a C_max_ of 37.56 µg/mL at 1 h post-dose after oral administration of 125 mg/kg, confirming its rapid absorption and systemic distribution. Notably, the presence of CuIIA in plasma was documented for the first time, along with the pharmacokinetic profiles of apigenin, phloretin, CuB, CuE, and CuI. In parallel, the segregant H387 M16 was characterized via colorimetric assays, thin-layer chromatography (TLC), HPLC, and antioxidant activity tests, which revealed high levels of flavonoids, phenols, and cucurbitacins, with an antioxidant activity of approximately 75% at the highest tested dose (1 mg/mL), supporting its suitability for future bioassays. Overall, these findings not only provide novel pharmacokinetic data for key metabolites of the H387 07 hybrid but also establish the phytochemical and antioxidant profile of its segregant H387 M16. This dual characterization strengthens the evidence of the therapeutic potential of *Sechium* genotypes and provides a valuable foundation for future studies aiming to develop standardized protocols and explore translational applications in drug development and natural product-based therapies.

## 1. Introduction

*Sechium edule* (Jacq.) Sw. (Cucurbitaceae), commonly known as chayote, christophene, vegetable pear, or chocho, is a species native to Mesoamerica whose use dates back to pre-Columbian times. Mexico is recognized as one of the main centers of diversity for this species [1]. Pharmacological studies have documented its diverse biological activities; its antibacterial activity has been demonstrated by Sulistiyani et al. [2], who reported that extracts inhibited the growth of *Staphylococcus aureus*, *Bacillus subtilis*, *Pseudomonas aeruginosa*, and *Escherichia coli*, with the ethyl acetate extract being the most active, an effect attributed to the phenol, flavonoid, and terpenoid contents. Regarding its antiulcer activity, in albino rat models with pylorus ligation and ethanol-induced ulcers, ethanolic extracts of *Sechium edule* fruit (100, 300, and 500 mg/kg, orally) significantly reduced the number of gastric petechiae, with inhibition percentages of 25.00%, 52.08%, and 70.83%, respectively. These results demonstrate a gastroprotective effect comparable to that of ranitidine (100 mg/kg) [3]. Concerning its antioxidant effects, Loizzo et al. [4], reported that the fresh pulp exhibited strong activity in the 2,2′-azino-bis(3-ethylbenzothiazoline-6-sulfonic acid) (ABTS) assay with a half maximal inhibitory concentration (IC_50_) of approximately 0.1 mg/mL, while peel showed greater activity in the 2,2-diphenyl-1-picrylhydrazyl (DPPH) test (IC_50_ ≈ 0.4 mg/mL), correlating with high levels of phenols, flavonoids, carotenoids, and vitamin C across peel, leaves, and pulp. Its hypoglycemic potential was demonstrated by Siahaan et al. [5], who showed that oral administration of ethanolic fruit extracts (100–200 mg/kg) in streptozotocin-induced hyperglycemic mice significantly reduced blood glucose levels over 28 days and promoted β-cell restoration, possibly mediated by flavonoid antioxidant mechanisms. Its antihypertensive effect has been reported by Islami et al. [6], who demonstrated that daily intake of *S. edule* juice significantly reduced systolic and diastolic blood pressure in postpartum women. In addition, its nephroprotective properties were described by Mumtaz et al. [7] aqueous leaf extracts (200 mg/kg) lowered serum creatinine, urea, and uric acid; increased total protein; and improved renal histology in nephrotoxic and diabetic models. Finally, the anticancer potential of *S. edule* has been observed; Cadena-Iñiguez et al. [8] demonstrated that methanolic extracts from different *S. edule* varieties exerted antiproliferative effects against several tumor cell lines, including mouse lung fibrosarcoma (L929), human cervical carcinoma (HeLa), and mouse macrophage leukemia (P388), with activity varying among varieties.

Building on these findings, special attention has been given to uncovering the secondary metabolites responsible for these effects. Cucurbitacins, a group of highly oxygenated tetracyclic triterpenoids characteristic of the Cucurbitaceae family, are among the most bioactive compounds in *S. edule*. Together with polyphenols (Supplementary Figure S1), these metabolites are considered the main contributors to its pharmacological properties. They have demonstrated diverse biological activities such as anti-inflammatory [9,10], hepatoprotective [11,12], antimicrobial [13,14], antioxidant [15,16], anthelmintic [17,18], antiviral [19,20], antihyperglycemic [21,22], and cardioprotective effects [23,24]. However, their most studied property is their anticancer activity, with mechanisms involving autophagy induction, pro-apoptosis, and inhibition of cell proliferation [25,26,27], making them relevant targets for phytochemical and pharmacokinetic studies.

In recent years, new genotypes with inedible characteristics have been developed, such as the hybrid *Sechium* H387 07 (*S. edule var. albus levis* × *S. edule amarus sylvestris*), through breeding programs [28]. Their inedibility is mainly attributed to the accumulation of secondary metabolites, particularly cucurbitacins, which impart an intense bitterness and render the fruits unsuitable for consumption [29]. The hybrid H387 07 has shown the ability to induce apoptosis in murine leukemia cell lines P388 (macrophage) and J774 (monocytic) and the myelomonocytic leukemia cell line WEHI-3 [30]. Despite extensive bioactivity studies, pharmacokinetic profiles of H387 07 and detailed phytochemical data for H387 M16 remain limited. Addressing these gaps is essential for clarifying their therapeutic potential and for guiding future applications in natural product-based drug development.

This study aimed to expand our understanding of *Sechium* variants by characterizing the segregant H387 M16, derived from H387 07, through preliminary phytochemical colorimetric tests (for phenols, flavonoids, terpenoids, saponins, alkaloids, and tannins), as well as TLC, HPLC, and antioxidant capacity assays. To elucidate the chemical basis of the therapeutic potential of *Sechium edule* genotypes, we first characterized the phytochemical profiles of the H387 07 hybrid and its segregant H387 M16 using qualitative and quantitative assays, as described below. In the experiments, the H387 07 hybrid was selected for a pharmacokinetic study in mice because it has exhibited a higher cucurbitacin content and stronger antiproliferative activity in earlier assays. This means that the likelihood of successfully detecting these metabolites in biological samples is increased. The H387 M16 segregant was not tested in vivo in our analysis due to the limited quantity of extract available and its intended use as a reference for comparative phytochemical analysis.

## 2. Results and Discussion

### 2.1. Phytochemical Characterization of Sechium H387 07 Hybrid and Segregants

Preliminary qualitative phytochemical screening of methanolic extracts of *Sechium* H387 07 and H387 M16 revealed the presence of saponins, flavonoids, phenols, and terpenoids (including cucurbitacins B, D, I, and E) and the absence of alkaloids in both genotypes. Tannins were detected only in H387 07. Preliminary tests were conducted on methanolic extracts obtained from the dried fruit of each genotype, using standard colorimetric assays for phenols, flavonoids, terpenoids, alkaloids, tannins, and saponins. The qualitative abundance of each metabolite group was recorded as intense (+++), moderate (++), weak (+), or absent (−) based on comparing the relative intensities of the colorimetric reaction or TLC spot with positive controls. In this scale, “+++” represents the highest observable intensity, “++” represents a clearly visible but moderate intensity, and “+” represents a faint but detectable signal. The results are summarized in Table 1. Phenols and terpenoids were strongly present in H387 07 (+++), whereas flavonoids showed a weaker presence (+) in both genotypes. Saponins were moderately present (++) in the H387 M16 extract and weakly present (+) in H387 07.

The observed phytochemical profile aligns well with previous reports for *Sechium edule*, where flavonoids, tannins, and terpenoids (including cucurbitacins) are common constituents across genotypes [31]. Although flavonoids were only weakly detected in the present screening, they were further explored because previous studies have consistently identified them as relevant bioactive compounds in *S. edule*, particularly in relation to their antioxidant and antiproliferative properties [32,33]. This justifies their inclusion in subsequent chromatographic analyses, despite their lower qualitative abundance in our preliminary tests. Moreover, genotype–environment interactions appear to be a major driver of variability in the secondary metabolite content, as demonstrated in studies on cowpea and other Cucurbitaceae, where flavonoids and tannins were shown to significantly vary depending on the environmental conditions and genotype [34]. The exclusive presence of tannins in *Sechium* H387 07 underscores this intra-specific variation and may reflect adaptive traits shaped by environmental or domestication influences. Such differences are relevant when selecting segregants for targeted pharmacological evaluation.

#### 2.1.1. Flavonoids Identified

Methanolic extracts of *Sechium* H387 07 and H387 M16 contained flavonoids such as rutin, morin, quercetin, catechin, hesperidin, phloridzin, naringenin, and phloretin. Catechin predominated in both genotypes (0.09205 and 0.14132 mg/g, respectively), with a higher total flavonoid concentration in H387 M16 compared to the hybrid H387 07 (Table 2). Similarly to the findings of Aguiñiga-Sánchez et al. [32], the presence of myricetin, quercetin, naringenin, apigenin, and phloretin was confirmed in the *Sechium* H387 07 hybrid extract; these are compounds that are strongly associated with antioxidant activity [32]. Likewise, Aguiñiga-Sánchez et al. [35] described secondary metabolites such as flavonoids, terpenes, and tannins in this hybrid, reporting higher concentrations compared to other *Sechium* variants.

In our analysis, phenols and saponins were more abundant in the *Sechium* H387 07 extract, while HPLC analysis revealed a higher flavonoid content in the segregant H387 M16 extract, accompanied by a lower amount of cucurbitacins. This contrasting distribution of secondary metabolites may reflect underlying genetic differences between the hybrid and its segregant, which can lead to shifts in biosynthetic pathway activation and result in variable accumulation of flavonoids compared to cucurbitacins. Such genotype-dependent phytochemical variation has been documented in other plant systems, in which genetic diversity significantly shapes metabolic profiles and biological activities [36]. Furthermore, even closely related genotypes can display markedly different chemical phenotypes, underscoring the evolutionary lability of plants’ secondary chemistry [37]. These patterns may stem from genotype-by-environment interactions that modulate phenotypic plasticity, influencing metabolite synthesis in response to external stimuli [38]. For example, the elevated content of cucurbitacins in the *Sechium* H387 07 extract may contribute to its previously reported antiproliferative effects. These findings highlight how subtle genetic differences between closely related genotypes can confer significant functional divergence through distinct phytochemical profiles.

#### 2.1.2. Cucurbitacins Identified

The methanolic extract of *Sechium* H387 07 exhibited the highest total concentration of cucurbitacins (8.25 mg/g), more than threefold greater than that detected in the segregant H387 M16 extract (2.47 mg/g; Table 3). Interestingly, the cucurbitacin concentration in the H387 07 hybrid was approximately 2.5 times higher than that previously reported by Aguiñiga-Sánchez., 2013 [35] (3.305 mg/g), suggesting that environmental or methodological factors may influence the metabolite yield. Among the identified compounds, CuB was predominant in both the hybrid and its segregant. This compound is one of the most extensively investigated cucurbitacins because of its wide range of pharmacological activities, notably its anticancer properties.

In vitro and in vivo studies have demonstrated that CuB exerts strong antiproliferative effects through the inhibition of diverse tumor cell lines [39,40] and through its antimicrobial, antiviral [41], anti-inflammatory [42], anti-aging [43], antidiabetic [44], anti-hypertrophic, and antifibrotic properties [24]. Its actions as a protector of mouse memory in Amyloid Precursor Protein/Presenilin 1 (APP/PS1) and as a neurogenesis inducer have also been described [45]. Therefore, the predominance of CuB in *Sechium edule* fruits underscores the pharmacological relevance of this hybrid and its segregant, as well as the importance of their phytochemical characterization for potential biomedical applications.

#### 2.1.3. Identification of Cucurbitacins by High-Performance Thin-Layer Chromatography

High-Performance Thin-Layer Chromatography (HPTLC) analysis of H387 07 and H387 M16 extracts revealed the presence of cucurbitacins CuB, CuD, CuI, CuE, and CuIIA. Importantly, although CuIIA was not detected in the initial HPLC analysis of the extracts, most likely because its concentration was below the detection limit or overlapped with other chromatographic peaks, it was clearly identified by HPTLC using reference standards and later confirmed in vivo after oral administration of the *Sechium* H387 07 extract. In HPTLC, direct application of the extract onto the plate and exposure to anisaldehyde sulfuric acid under UV light facilitated the visualization of the corresponding band in parallel with the standards. The presence of CuIIA was subsequently confirmed in serum in the in vivo experiments.

**Figure 1:**
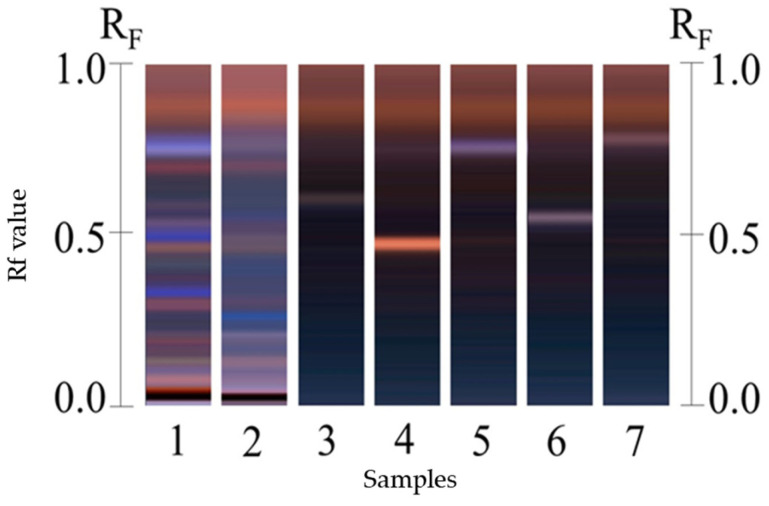
High-Performance Thin-Layer Chromatography (HPTLC) analysis of cucurbitacins in methanolic extract of *Sechium* H387 07 and H387 M16. Lane 1: H387 M16; Lane 2: H387 07; Lane 3: CuI standard; Lane 4: CuIIA standard; Lane 5: CuB standard; Lane 6: CuD standard; Lane 7: CuE standard. Blue bands indicate cucurbitacin presence, with the CuB band at retention factor (Rf) 0.41 clearly visible in both genotypes.

Notably, the H387 M16 extract showed higher levels of all the cucurbitacins. Chromatography also revealed differences in the retention factor (Rf) of some bands, suggesting differences in cucurbitacin composition among genotypes. For example, the *Sechium* H387 07 extract exhibited a blue band with a surrounding Rf that was not identified in H387 M16 (Figure 1, lanes 1 and 2). Although multiple blue bands were present, they were less prominent in H387 M16. The higher concentration observed in H387 M16 could be explained by the expression of latent traits transmitted by one of its parents through processes such as genetic recombination or domestication, since variability in secondary metabolite concentrations is often linked to adaptive responses to the environment [46]. In this case, hybridization may not only trigger quantitative differences in cucurbitacins but also promote the expression of phenotypic traits that are usually latent in their parents. Such traits can emerge as a result of environmental and internal interactions, potentially inducing phenotypic characteristics within a short period of time, even in evolutionarily divergent species [47].

The data on the presence and concentration of metabolites obtained in our analysis is valuable for comparison with other segregants. Although there are reports on the biological effects of various *Sechium* genotypes, their application based on phytochemical composition has not been systematically addressed, making the reproducibility of their effects difficult [48]. Furthermore, phytochemical descriptions of these materials provide a useful framework for evaluating their anticancer effects, since identifying these metabolites brings us closer to understanding the causal agents underlying the anticancer effects previously reported for this extract. However, some studies have also demonstrated a greater potential of crude extracts of the *Sechium* H387 07 hybrid when they are not fractionated or isolated [49]. With the confirmation of cucurbitacins and other metabolites in H387 07 and H387 M16 extracts via HPTLC, we subsequently evaluated the antioxidant capacity of these extracts using the DPPH assay to assess their potential protective effects against oxidative stress.

### 2.2. Antioxidant Capacity of the Extracts by 2,2-Diphenyl-1-Picrylhydrazyl

Both H387 M16 and *Sechium* H387 07 extracts showed dose-dependent DPPH inhibition effects, with their activities increasing from 0.015 to 1.00 mg/mL. At the highest tested concentrations (1 mg/mL), both genotypes achieved inhibition levels close to 80%. The *Sechium* H387 07 hybrid displayed a higher efficacy, with an IC_50_ of 0.1178 mg/mL, compared to the segregant H387 M16 (IC_50_ = 0.3471 mg/mL). The mean inhibitory dose for the *Sechium* H387 07 hybrid extract was 0.6 mg/mL; the segregant H387 M16 extract exhibited an antioxidant activity of approximately 75% at the highest concentration tested (1 mg/mL).

**Figure 2:**
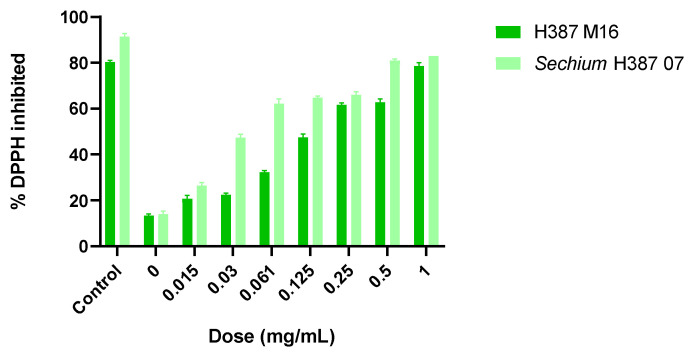
Percentage of 2,2-Diphenyl-1-picrylhydrazyl (DPPH) inhibition by methanolic extracts of *Sechium* H387 07 and H387 M16. Inhibition values were measured at concentrations ranging from 0.015 to 1.00 mg/mL. Bars represent means ± SD of triplicate measurements. Different colors are used to differentiate genotypes.

These findings corroborate previous observations reported by Aguiñiga-Sánchez et al. [32], who described similar inhibitory effects for the *Sechium* H387 07 hybrid (IC_50_ = 0.88 mg/mL, 70% inhibition). However, unlike this earlier study, the present work extends the analysis by directly comparing the hybrid with its segregant, H387 M16, under the same experimental conditions. This comparative approach highlights the consistently lower IC_50_ of the hybrid, while also revealing that the segregant maintains a substantial inhibitory capacity (~75%), supporting its potential as a candidate for future in vitro bioassays (Figure 2).

Having established the phytochemical profiles of H387 07 and H387 M16, which revealed high levels of bioactive cucurbitacins and flavonoids, we next investigated the pharmacokinetic behavior of H387 07 extracts in mice to assess how these metabolites were absorbed, distributed, and metabolized in vivo, as detailed in the following sections.

### 2.3. Determination of Cardiac, Splenic, Hepatic, Renal, and Cerebral Indices in Mice Administered with the Sechium H387 07 Extract

To advance the pharmacological characterization of *Sechium* genotypes, in vivo analyses in this study were focused exclusively on the *Sechium* H387 07 hybrid extract. The segregant H387 M16 was not administered in vivo due to the limited yield obtained, which restricted its use to phytochemical characterization. In addition, *Sechium* H387 07 was prioritized for pharmacokinetic evaluation because its bioactivity had already been demonstrated in previous in vitro assays, providing a stronger basis for systemic testing. In vivo studies with H387 M16 are planned as part of future work, once sufficient material is available. Accordingly, to assess whether the treatment had a significant effect on the organs, the organ index was calculated as the organ weight-to-body weight ratio for each dose and sampling time. For this analysis, samples were collected at 0, 0.5, 1, 2, 3, 6, 12, 24, and 48 h post-treatment.

The results obtained using analysis of variance (ANOVA) with factorial arrangement did not show significant differences between doses or treatments. Likewise, the interaction between these factors was also not significant (*p* > 0.05) (Supplementary Figure S2). However, when considering the total weight of all organs, significant differences (*p* < 0.05) were observed among the treatments. The 250 mg/kg dose presented the highest weight compared to phosphate-buffered saline (PBS) treatments of 37.875 g and 33.550 g (*p* < 0.05). These results differ from those previously reported [35], where a significant increase in the size of the thymus and spleen was observed in mice administered 8, 250, 500, and 1000 mg/kg doses of extract via intraperitoneal and gastric routes. By contrast, in our analysis, oral administration of the extract did not cause alterations in the size of the brain, heart, lungs, kidneys, or liver. This observation suggests that the extract of *Sechium* H387 07, when administered at appropriate oral doses, may be better tolerated and less likely to induce alterations in vital organs, which is a relevant consideration for its potential therapeutic application.

### 2.4. Polyphenols in Serum, Liver, and Urine

**Figure 3:**
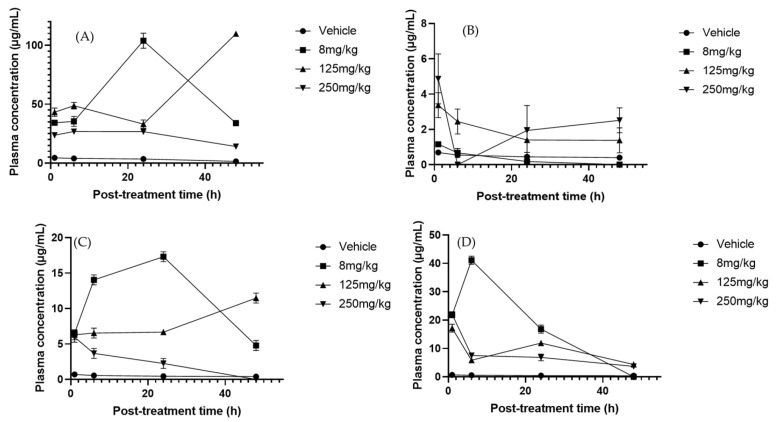
Plasma concentration–time profiles of flavonoids after oral administration of *Sechium* H387 07 extract at doses of 8, 125, and 250 mg/kg. (**A**) Rutin, (**B**) phloretin, (**C**) morin, (**D**) catechin. Vehicle-treated animals (phosphate-buffered saline, PBS) are shown for comparison. Data are expressed as means ± SEM (*n* = 4 per group).

Multiple regression analysis revealed significant changes in serum polyphenol concentrations depending on treatment and exposure times. Rutin was the most affected metabolite, showing a marked decrease at the 250 mg/kg dose (Figure 3A). No significant effects were observed with changes in treatment duration, possibly due to accelerated metabolism or excretion at higher concentrations [50]. However, this could also reflect increased uptake of rutin by immune cells, since Ganeshpurkar et al. [51] demonstrated that rutin exerts immunomodulatory effects by reducing inflammatory mediators such as tumor necrosis factor-α (TNF-α), interleukin-1β (IL-1β), interleukin-6 (IL-6), and nitric oxide (NO) in lipopolysaccharide (LPS) macrophages. This suggests that part of the decline in plasma levels may be explained by the cellular assimilation of rutin during the modulation of inflammatory responses. In contrast, naringenin remained stable across treatments and exposure times, likely explained by its high affinity for human serum albumin (HSA) and conjugates such as glucuronide sulfates, which delays clearance and leads to steady plasma levels [52,53]. Phloretin, although not significantly present in serum, decreased between 6 and 24 h at 125 mg/kg (Figure 3B), suggesting rapid elimination through the kidneys. Previously, Crespy et al. [54] reported that after feeding mice with a diet rich in this compound, no significant levels were observed in plasma at 10 h post-treatment; at 24 h, the levels returned to basal levels, suggesting that phloretin is substantially eliminated through urine. Similarly, Wang et al. [55] found that phloretin is rapidly absorbed and eliminated from the plasma post-oral administration, which corroborates the possibility of efficient renal elimination. Similarly, morin significantly declined under the 250 mg/kg treatment (Figure 3C), reflecting a dose-dependent response that may result from reduced albumin binding at higher concentrations, facilitating metabolism [56]. Although morin has been reported to have a high affinity for serum albumin [57], it is likely that at high concentrations, the binding decreases, increasing its capacity to be metabolized. By contrast, apigenin significantly increased with the 125 mg/kg doses at 24 and 48 h, consistent with its relatively slow elimination and tendency to accumulate in serum [58,59]. Catechin, in turn, decreased progressively at both 8 and 250 mg/kg (Figure 3D), a pattern associated with rapid metabolism mediated by catechol-O-methyltransferase [60], likely involving methylation by catechol-O-methyltransferase, since catechin is a direct substrate of this enzyme [61]. This may have led to its reduction. Collectively, these findings underscore the influence of both the treatment concentration and exposure time on serum flavonoid dynamics.

**Figure 4:**
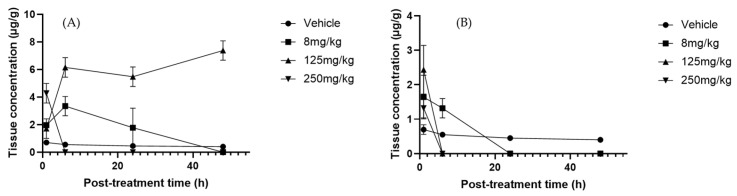
Liver concentration–time profiles of (**A**) phloretin and (**B**) naringenin after oral administration of *Sechium edule* H387 07 extract in mice at doses of 8, 125, and 250 mg/kg. Vehicle-treated animals (PBS) are shown for comparison. Data are expressed as means ± SEM (*n* = 4 per group).

In liver samples, phloretin and naringenin metabolites showed the most significant changes in response to time and treatment alterations. Naringenin significantly decreased after 24–48 h with the 8 mg/kg dose (*p*-value = 0.0141) (Figure 4B). This could be due to the accelerated hepatic metabolic activity, which leads to the more rapid breakdown of naringenin over time. The metabolic response of the liver could adjust to the presence of this compound, resulting in greater enzymatic activity that in turn leads to reduced naringenin levels [62], consistent with the induction of hepatic conjugating enzymes such as UDP-glucuronosyltransferase 1A1 (UGT1A1) and sulfotransferase 1A1 (SULT1A1) that accelerate its metabolism [63,64]. In contrast, phloretin increased significantly with a dose of 125 mg/kg at 6 and 48 h post-treatment (Figure 4A), indicating efficient absorption or delayed metabolism at this dose. The variation in concentrations could also be related to the liver’s ability to regulate and balance flavonoid levels depending on the duration of exposure [65].

Regarding urinary excretion, most polyphenols remained at basal concentrations and did not exhibit significant changes due to treatment. The only exception was hesperidin, which increased under the 8 mg/kg dose at 6 and 24 h (Supplementary Figure S3). This pattern suggests that renal elimination of polyphenols may occur through mechanisms independent of ABC transporters, which are commonly involved in flavonoid excretion. Given the stability of the basal concentrations and the lack of post-treatment changes in most metabolites, it is likely that their elimination is not only dependent on acute modulation of ABC transporters, but also involves processes such as glomerular filtration and active transport through renal tubule transporters, including MRP2 and BCRP. This is because some of these transporters can become saturated at high concentrations [66]. These results indicate that hesperidin is particularly sensitive to treatment modulation, while most polyphenols follow stable renal excretion pathways.

### 2.5. Identification of Cucurbitacins in the Serum, Liver, and Urine of Mice Treated with Sechium H387 07 Extract

**Figure 5:**
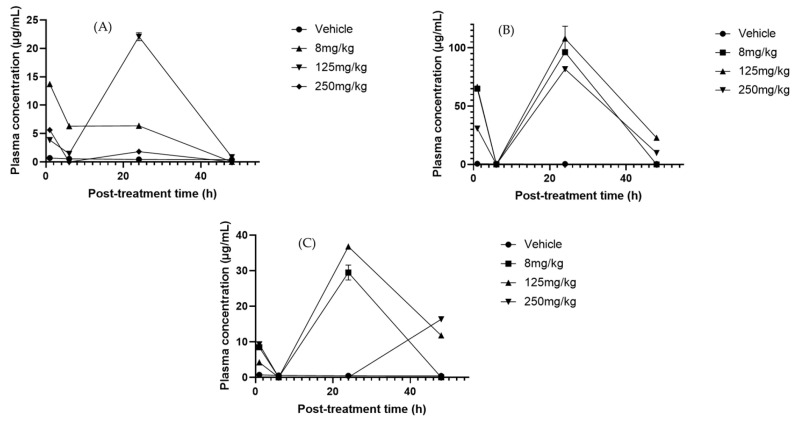
Plasma concentration–time profiles of cucurbitacins after oral administration of *Sechium* H387 07 extract at doses of 8, 125, and 250 mg/kg. (**A**) Cucurbitacin D (CuD), (**B**) cucurbitacin IIA (CuIIA), and (**C**) cucurbitacin B (CuB). Vehicle-treated animals (PBS) are shown for comparison. Data are presented as means ± SEM (*n* = 4 per group).

HPLC analysis of serum revealed the presence of CuB, CuD, CuE, and CuIIA, each showing distinct pharmacokinetic behaviors. CuD reached its C_max_ at 24 h with the 125 mg/kg dose (Figure 5A). Its pharmacokinetics remain poorly studied, making this result particularly relevant. In the liver, no significant variations in CuD, CuI, and CuE were consistently detected across treatments and time points (Supplementary Figure S4), suggesting stable hepatic metabolism and sustained tissue retention [67]. For CuI, no clear T_max_ was identified in serum, and concentrations remained near the limit of quantification, whereas in the liver, they stayed consistently high (>20 µg/mg at 125 and 250 mg/kg). This stable profile suggests a sustained hepatic retention of CuI rather than a clear elimination phase, which may indicate slow turnover or redistribution within liver tissue. CuIIA, in contrast, displayed a serum T_max_ of 24 h across all doses (Figure 5B). This differs from previous reports that described much faster absorption (~20 min) [68]. Interestingly, the serum profile showed a transient decrease to near-zero concentrations at 6 h, followed by a marked increase at 24 h, particularly evident at higher doses. Such a biphasic pattern may reflect reabsorption processes and is consistent with enterohepatic recirculation. Moreover, liver concentrations of CuIIa remained significant for up to 48 h with the 250 mg/kg treatment. This sustained level indicates that CuIIA may undergo enterohepatic circulation and metabolism in phases I and II. In addition, high concentrations were observed in urine (Supplementary Figure S6). It has been suggested that the kidney possibly plays a relevant role in the CuIIA metabolism; however, it is speculated that its metabolites could be involved in its efficiency [69].

CuB is one of the most relevant cucurbitacins because of its potential anticancer effects [70]. In our analysis, CuB was one of the most abundant components in blood. The anticancer capacity repeatedly observed in the extract of the *Sechium* H387 07 hybrid has been linked to the presence of these compounds [30]. The T_max_ of CuB was 24 h at a dose of 125 mg/kg (Figure 5C). Other studies have reported a T_max_ of up to 3 h at 8 mg/kg with a C_max_ of 3.41 × 10^−5^ µg/mL. In contrast, in this study, the C_max_ reached 37.27 µg/mL at 24 h. Hunsakunachai et al. [71] recorded up to 0.031 µg/mL of CuB in *Trichosanthes cucumerina* extracts, and Xiao et al. [72] observed a high concentration of CuB in systemic circulation (0.034 µg/mL) after a dose of 1 mg/kg was administered, higher than that observed at an eight-times higher dose. These differences suggest that the pharmacokinetic behavior of CuB may strongly depend on the extract matrix and co-occurring metabolites, which could explain the markedly higher concentration observed in the *Sechium* H387 07 extract.

Regarding urinary excretion, most cucurbitacins remained low and unaffected by treatment or exposure time. The main exception was CuE, which showed a clear increase under the 8 mg/kg treatment (Supplementary Figure S5). At higher or lower concentrations than those used in this study, absorption could be less efficient due to factors such as transporter saturation or activation of elimination mechanisms. On the other hand, CuE could be metabolized more efficiently at these concentrations, resulting in higher detectable levels in urine. Higher concentrations can activate alternative metabolic pathways that do not produce the same amount of excretable CuE. Indeed, pharmacokinetic studies indicate that CuE displays rapid clearance, with a plasma half-life in the range of 58–72% following oral dosing (100–200 µg/kg). Cucurbitacins also generally exhibit a low oral bioavailability (10%), with extensive tissue distribution and minimal excretion of the unmetabolized form, suggesting efficient biotransformation prior to elimination [73].

**Figure 6:**
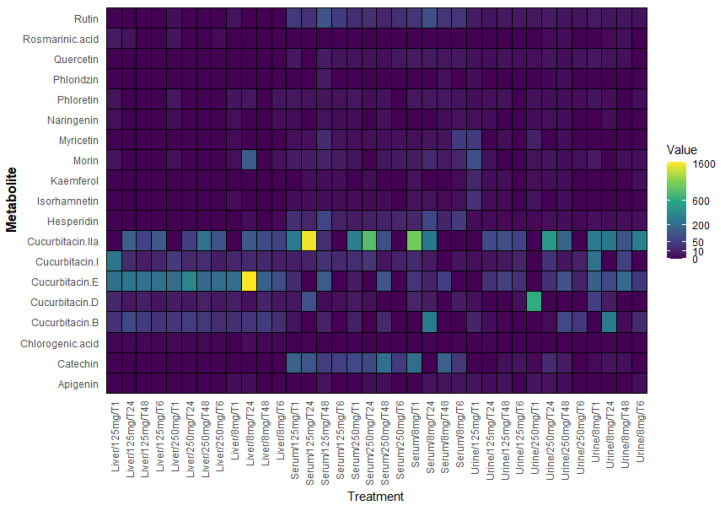
Heat map showing the relative concentration of secondary metabolites detected in serum (µg/mL), liver (µg/mg), and urine (µg/mL) samples of mice treated with *Sechium edule* H387 07 extract. The samples corresponded to three extract concentrations (8, 125, and 250 mg/kg) and four post-treatment sampling times (1, 6, 24, and 48 h). The color intensity represents the relative abundance of each compound, with yellow indicating the highest concentration and purple the lowest concentration.

In order to visualize the overall distribution of secondary metabolites across matrices, a heat map was generated (Figure 6). To ensure reliability, only metabolites with concentrations above the detection threshold (>2 µg/mL) were included in this analysis. This heat map highlights the higher accumulation of cucurbitacins and certain flavonoids in liver samples compared with serum and urine samples, providing a global perspective before each metabolite is analyzed individually.

Following the detection of polyphenols and cucurbitacins in serum, liver, and urine, and the assessment of organ indices in mice treated with H387 07 extracts, we quantified the pharmacokinetic parameters to characterize the in vivo disposition of these bioactive compounds.

### 2.6. Pharmacokinetic Analysis

**Figure 7:**
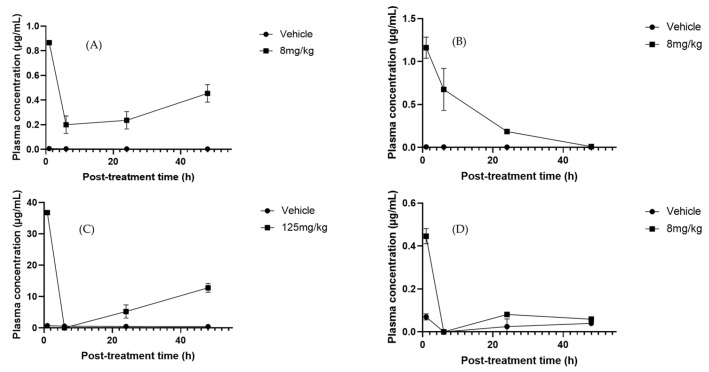
Plasma concentration–time profiles of selected metabolites after oral administration of *Sechium* H387 07 extract in mice. (**A**) Apigenin (8 mg/kg), (**B**) phloretin (8 mg/kg), (**C**) cucurbitacin B (CuB, 125 mg/kg), and (**D**) cucurbitacin E (CuE, 8 mg/kg). Vehicle-treated animals (PBS) are shown for comparison. Data are presented as means ± SEM (*n* = 4 per group).

To determine pharmacokinetic parameters, the most reliable results were obtained at doses that reached adequate concentrations for calculation after administration of the *Sechium* H387 07 extract at 2, 6, 24, and 48 h, as illustrated by apigenin (Figure 7A). This flavonoid is widely distributed in plants and has been described as having efficient intestinal absorption, particularly in the duodenum [74]. In our study, apigenin exhibited a T_1/2_ of greater than 5.6 h, notably longer than the 1.8–4.2 h reported in rats [75]. This suggests slower elimination in our experimental model, possibly due to formulation or matrix effects delaying metabolism. In this study, oral administration of the *Sechium* H387 07 extract yielded an area under the curve AUC_0−t_ of 2.12 μg·h/mL, which is slightly higher than the values reported by Chen et al. [76], for apigenin-7-O-β-D-glucoside after intravenous administration in rats (AUC_0−t_ ranging from 0.68 to 1.56 μg·h/mL depending on the dose). Differences may reflect differences in compound forms (glycoside vs. aglycone), matrix effects, or administration routes (oral versus intravenous). The estimated volume of distribution (V_d_) suggested extensive tissue distribution, while the relatively low clearance (0.82 L/h/kg) supports moderate elimination. These findings align with the report of long terminal half-lives and low clearances in rats [59]. In contrast, Walle et al. [77] observed much shorter elimination times in humans, reflecting species differences in absorption, metabolism, and clearance. They described extensive tissue distribution and enterohepatic recycling, contributing to a prolonged systemic presence despite low plasma concentrations. Overall, apigenin exhibited moderate absorption, a wide distribution, and slow clearance, features that suggest prolonged systemic exposure, possibly influenced by the matrix effects of the *Sechium* extract rather than rapid elimination, as reported in other models.

Phloretin exhibited higher systemic exposure (C_max_ = 1.12 µg/mL) and an AUC_0 ∞_ of 6.1 µg·h/mL. Its T_max_ was 4.2 h, shorter than that of apigenin, and its V_d_ (0.95 L/kg) indicated a more limited peripheral distribution. Its clearance (0.82 L/h/kg) was similar to that of apigenin, suggesting that the higher exposure of phloretin is mainly due to its higher absorption and plasma concentration (Figure 7B). Previous studies reported extensive tissue distributions in the liver, kidneys, lungs, and heart, but low blood–brain barrier penetration and relatively low clearance [78]. Together, these findings point to a good oral bioavailability and potential systemic retention, favorable for therapeutic applications.

Cucurbitacins are among the most extensively studied metabolites due to their medicinal properties, particularly their anticancer activity [79]. Among this group, CuB is one of the most investigated because of its diverse biological effects [70]. In our study, CuB exhibited a C_max_ of 37.56 µg/mL (T_max_ = 1 h), an AUC_0_–∞ of 2.05 µg·h/mL, and a T_max_ of 7.2 h (Figure 7C). Its high V_d_ (5.0 L/kg) suggests extensive tissue uptake, while its low clearance (0.70 L/h/kg) supports slow elimination. After an initial decline at 6 h, a secondary rise was observed at 24–48 h, indicating enterohepatic circulation, a well-documented pharmacokinetic mechanism whereby compounds excreted into bile are reabsorbed from the gut, producing multiple peaks and prolonging systemic presence [80]. In contrast, Hunsakunachai et al. [71] reported much lower systemic exposure in rats, with C_max_ values of 0.0097 µg/mL at 2 mg/kg and 0.0312 µg/mL at 4 mg/kg after oral administration of CuB from *Trichosanthes cucumerina*. These markedly lower concentrations, compared with the 37.56 µg/mL observed in our study at 125 mg/kg, highlight the poor oral bioavailability previously described for CuB. On the other hand, CuE showed a distinct profile, exhibiting a C_max_ of 0.42 µg/mL at T_max_ of 2.5 h, with an AUC_0 ∞_ of 1.80 µg·h/mL (Figure 7D). The high volume of distribution (4.8 L/kg) indicates extensive tissue distribution, while the moderate clearance rate (0.65 L/h/kg) reflects slower elimination than CuB. Previous studies have reported much lower peak plasma concentrations of approximately 0.009 µg/mL at 2 h post-dose and a longer plasma half-life, possibly associated with protein binding [55,81]. Despite discrepancies in systemic exposure, our data support the hypothesis that cucurbitacins exhibit strong tissue affinity and variable pharmacokinetics depending on the extract matrix and experimental model.

Overall, the pharmacokinetic parameter values obtained in this study indicate that the flavonoids apigenin and phloretin are characterized by moderate absorption and limited distributions, whereas CuB and CuE display wider tissue distributions and slower elimination. This contrasting behavior highlights differences in chemical structures and physicochemical properties and may also reflect the complexity of the *Sechium* H387 07 hybrid extract. In this context, potential synergistic interactions among its metabolites could modulate absorption, enhance tissue uptake, and ultimately influence the overall bioavailability profile.

## 3. Materials and Methods

### 3.1. Plant Material

The fruits used in this study were obtained from the National Germplasm Bank of *Sechium* in Mexico (19° 08′ 48″ N and 97° 57′ 00″ W) and presented a horticultural maturity of 18 ± 2 days after anthesis [82]. The vegetative material used was the hybrid *Sechium* H 387 07; this genotype was formed by controlled crossing of accession 290-05 of the *virens levis* varietal group of the species *Sechium edule* (Jacq.) Sw. as the male parent (pollen donor) and accession Madre Negra (273-05) of the *amarus sylvestris* varietal group of the species *Sechium edule* (Jacq.) Sw as the female donor (receiver). The segregant H387 M16 is derived from the same parental cross but represents a non-stabilized lineage, displaying variable phenotypic and phytochemical traits compared to the stable hybrid. For this reason, H387 M16 was considered a contrasting genotype for phytochemical characterization in this study.

The material was washed, dehydrated, and ground. Dehydration was performed in an oven (BLUE-M, Electronic Company/Blue Island, IL, USA) at 40 °C. The dehydrated tissue was ground to a particle size of 2 mm (No. 10 mesh), and the samples were weighed to determine the yield of biological material.

### 3.2. Extract Preparation

The extracts were obtained from the hybrid *Sechium* H387 07 and the segregant H387 M16. The extraction method was discontinuous, using methanol, with 250 g of dry powder from *Sechium* H387 07 and 108 g from H387 M16. The tissue was macerated every 48 h at room temperature. The difference in the initial dry mass between the two genotypes was due to the availability of biological material at the time of collection, which limited the amount of H387 M16 that could be processed. The samples were filtered through filter paper (Whatman™, Whatman International Ltd., Kent, UK). In each extraction cycle, the cartridge was replaced, and extraction continued until the macerated residue became colorless. It was subsequently evaporated under a reduced negative pressure (vacuum) at 50 °C (Buchi Evaporator R-114, Buchi, Flawil, Switzerland). Crude methanolic extracts were then obtained after complete removal of the solvent. This procedure was repeated approximately 30 times.

### 3.3. Phytochemical Analysis by Preliminary Tests

For the preparation of the samples, 5 mL of 80% methanol in water (*v*/*v*) was added to 3 g of each extract from *Sechium* H387 07 and H387 M16. This mixture was kept in an ultrasonic bath for 15 min, left to stand for 5 min, and the ultrasound treatment was repeated for another 15 min. A positive and a negative control were included for each analysis.

The phytochemical profile of the fruits of *Sechium* H387 07 and H387 M16 was developed through qualitative analysis of the main groups of secondary metabolites in the extracts, including phenols, flavonoids, terpenoids, alkaloids, tannins, and saponins. This was achieved using a series of protocols established for this type of testing at the Phytochemistry Laboratory at the Colegio de Postgraduados. Specifically, phenols were identified using the Folin–Ciocalteu assay (with gallic acid, Sigma-Aldrich, St. Louis, MO, USA, as a positive control); flavonoids were detected using the Shinoda test (magnesium–HCl reaction, using quercetin, Sigma-Aldrich, as a reference); terpenoids were evaluated through the Liebermann–Burchard reaction (using ursolic acid, Sigma-Aldrich, as a control); alkaloids were tested with Dragendorff’s reagent (an authenticated *Lupinus* spp. alkaloid extract from the Phytochemistry Laboratory collection, Colegio de Postgraduados, was used as a positive control); tannins were detected with ferric chloride (standardized tannin extract, Sigma-Aldrich, as a control); and saponins were detected via foam formation in aqueous solution. In all cases, the negative control was 80% methanol (*v*/*v*), and qualitative intensity was recorded according to the colorimetric or TLC responses.

The relative abundance of each metabolite group was classified on a semi-quantitative scale commonly applied in preliminary phytochemical screening: intense (+++), moderate (++), weak (+), or absent (−). These categories were assigned based on the comparative intensity of the colorimetric reaction relative to the corresponding positive control, as has been described in standard phytochemical screening practices [83].

#### 3.3.1. Thin-Layer Chromatography Detection

Qualitative analysis of the secondary metabolite groups of the *Sechium* H387 07 and H387 M16 extracts was performed using TLC. Ten microliters of each sample was applied on a 10 × 10 cm silica plate, spaced 1 cm apart, with a 1 cm solvent front. To increase the concentration, the total volume (10 µL) was applied in four 2.5 µL aliquots at each site, allowing for drying between applications. Different solvent systems were used depending on the metabolite analyzed: ethyl acetate–methanol for tannins, dichloromethane–methanol–ammonium hydroxide for alkaloids, chloroform–methanol–water for flavonoids, chloroform–methanol for terpenoids, ethyl acetate–formic acid–acetic acid–water for saponins, and ethyl acetate–methanol for phenols. The chromatography chamber was allowed to saturate for 30 min before the silica plate was inserted into the samples. The presence of the compound groups in the chromatograms was detected with specific reagents for each compound, which were introduced into an oven previously heated to 105–110 °C for 5 min.

#### 3.3.2. Identification of Secondary Metabolites by High-Performance Liquid Chromatography

Flavonoids, phenolic acids, and cucurbitacins were identified by HPLC. Samples of 20 mg of the *Sechium* H387 07 and H387 M16 extracts were dissolved in 1 mL of HPLC-grade methanol (Sigma-Aldrich, USA) and filtered through a 0.22 µm membrane (Millipore, Cork, Ireland) before injection. Analyses were performed on an Agilent HPLC system (Agilent Technologies, Santa Clara, CA, USA) equipped with a photodiode array detector. Separation was achieved on a reverse-phase C18 column (250 × 4.6 mm, 5 µm).

The mobile phase consisted of solvent A (water with 0.1% formic acid) and solvent B (acetonitrile), using the following gradient program: 10% B at 0 min, increased to 60% B at 30 min, then to 90% B at 40 min, held for 5 min, and returned to initial conditions in 5 min. The flow rate was 1.0 mL/min, the injection volume was 20 µL, and detection wavelengths were set at 210, 254, 280, and 340 nm according to the maximum absorbance of each compound class. Representative chromatograms for each class of metabolites were obtained and compared with authentic standards.

Identification and quantification were based on comparison with authentic standards of flavonoids (rutin, quercetin, catechin, morin, hesperidin, phloridzin, naringenin, and phloretin), phenolic acids (chlorogenic, caffeic, ferulic, gallic, and vanillic acids), and cucurbitacins (B, D, E, I, and IIa), all purchased from Sigma-Aldrich (USA). Calibration curves were prepared for each standard using at least six concentration levels. Linearity was confirmed with correlation coefficients (R^2^) ≥ 0.995.

Accuracy and precision were determined using quality control samples at low, medium, and high concentrations. The lower limit of quantification (LLOQ) and upper limit of quantification (ULOQ) were established within the calibration curve range, with acceptance criteria for accuracy within ±15% (±20% for LLOQ) and for precision with a coefficient of variation (CV) ≤ 15% (≤20% for LLOQ). The limit of detection (LOD) and limit of quantification (LOQ) were calculated based on signal-to-noise ratios of 3:1 and 10:1, respectively. Specificity was confirmed by the absence of interfering peaks in blank samples.

#### 3.3.3. Procedure for Identification of Cucurbitacins by High-Performance Thin-Layer Chromatography

HPTLC analysis was used to evaluate the presence of cucurbitacins in the *Sechium* H387 07 and H387 M16 samples. Ten µL of each sample, at a concentration of 10 mg/mL in 80% aqueous methanol, was applied onto a chromatographic plate. The mobile phase consisted of chloroform–methanol (9:1, *v*/*v*), with a chamber saturation time of 20 min, and the solvent front was allowed to migrate 85 mm. The plate was stained with sulfuric acid–anisaldehyde at 100 °C for 3 min, and the presence of cucurbitacins was visualized under ultraviolet light at 366 nm.

## 4. Assessment of Antioxidant Capacity via 2,2-Diphenyl-1-Picrylhydrazyl Assay

The antioxidant capacity of the extracts was evaluated using DPPH radical scavenging method, which is a cell-free chemical assay commonly used to estimate the radical neutralizing activity of natural products. A 0.1 mM DPPH solution was prepared in methanol (Sigma-Aldrich, USA). The standard curve was obtained with concentrations of 0, 0.01, 0.02, 0.04, 0.06, 0.08, and 0.1 mM, and the concentrations of the evaluated extracts of *Sechium* H387 07 and H387 M16 were 0.15, 0.3, 0.61, 0.125, 0.25, 0.5, and 1 mg/mL. For each reaction, 50 µL of the extract solution was mixed with 150 µL of the DPPH solution in a 96-well ELISA plate. The mixtures were incubated for 30 min at room temperature in the dark with gentle shaking, and discoloration was measured spectrophotometrically at 517 nm.

Trolox (Sigma-Aldrich, USA) was used as a positive control at equivalent concentrations, while methanol served as the negative control. The percentage of DPPH inhibition was calculated using the following formula:

%DPPH = (A control − A sample) × 100/A control

where A control is the absorbance of the DPPH control solution and A sample is the absorbance of the extract or standard.

To determine the half-maximal inhibitory concentration (IC_50_), the percentage of inhibition was plotted against the logarithm of extract concentration, and nonlinear regression analysis was applied to fit a sigmoidal dose–response curve. The IC_50_ value was defined as the concentration of extract required to inhibit 50% of the DPPH radical, as calculated from the regression equation.

## 5. Animal Maintenance

Pharmacokinetic parameters were evaluated in female CD-1 mice (*Mus musculus* L.) provided by the vivarium of the Faculty of Higher Studies, Zaragoza, National Autonomous University of Mexico. All mice were clinically healthy, approximately 12 weeks old, and weighed ~30 g. Inclusion criteria were normal appearance, healthy behavior, and body weight within the 29–33 g range, whereas animals outside this range or exhibiting signs of illness were excluded. In accordance with the Mexican Official Standard NOM-062-ZOO-1999, which includes techniques for the production, care, and use of laboratory animals and international guidelines for laboratory animal research, initial pharmacokinetic studies are typically conducted on a single sex to reduce variability and obtain consistent results. Female mice were selected as the available cohort from the institutional vivarium, and their use allowed us to establish baseline pharmacokinetic parameters under standardized conditions. The animals were maintained under a 12 h light/dark cycle and at a room temperature of ~22 °C and were provided with sterile water and standard food ad libitum. Animals were randomly assigned to treatment groups. Treatment allocation and sample collection were blinded. All experimental procedures were approved by the Research Ethics Committee of the Faculty of Higher Studies, Zaragoza (FESZ/CEI/1/1/5/27/23).

### 5.1. Mouse Treatment

Mice (*n* = 4 per dose) were orally administered the hybrid *Sechium* H387 07 extract at doses of 8, 125, and 250 mg/kg. These doses were chosen based on previous in vivo studies with *Sechium* extracts [35], where the LD_50_ was reported to be higher than 1000 mg/kg and no organ toxicity was observed at doses below 500 mg/kg. Antitumor effects have also been detected at 50 mg/kg in murine models of acute myeloid leukemia and breast cancer. In this context, 8 mg/kg was chosen as a low reference dose, while 125 and 250 mg/kg were included as intermediate and high doses, respectively, to allow for the evaluation of both the pharmacological activity and tolerability of the extract. For the organ index evaluation, tissues were collected at 0, 0.5, 1, 2, 3, 6, 12, 24, and 48 h post-treatment, allowing for the assessment of early and late changes in organ weight. In contrast, for the pharmacokinetic analysis of secondary metabolites, blood and tissue samples were collected only at 1, 6, 24, and 48 h post-dose due to limited HPLC availability, which restricted the number of feasible sampling points. Small sample size may limit statistical power, warranting larger cohorts in future studies. In addition, the restricted number of sampling points due to HPLC availability constrains the precision of pharmacokinetic estimations, and interspecies differences should be considered when extrapolating these findings to humans.

Two control groups were included: one that did not receive the extract and another that received the vehicle (PBS). The segregant H387 M16 was not administered in vivo; this decision was based on previous reports identifying this genotype as a potential anticancer agent [30], which provided a solid rationale for evaluating its systemic effects. In contrast, the biological activity of the H387 M16 extract has not yet been fully characterized, and in vivo assessments of this extract are planned for future studies.

### 5.2. Pharmacokinetic Evaluation of Secondary Metabolites in Sechium H387 07

For the HPLC quantification of metabolites, concentrations were expressed as µg/mL for serum and urine and as µg/mg of tissue for liver samples. The results were translated into graphical form using the identified metabolites. The C_max_ and time to reach maximum concentration (T_max_) of the secondary metabolites were obtained directly from the concentration–time curve.

The area under the plasma concentration–time curve from time zero to the last measurable concentration ($AUC_{0-t}$) was calculated using the linear trapezoidal rule, according to the following formula:

$$AUC_{0-t}=\overset{n-1}{\underset{i=1}{\sum }}\frac{\left(C_{i}+C_{i+1}\right)}{2}\times \left(t_{i+1}-t_{i}\right)$$

where $C_{i}$ and $C_{i+1}$ are the plasma concentrations at consecutive time points $t_{i}$ and $t_{i+1}$. The total area under the curve ($AUC_{0-\infty }$) was obtained as the sum of $AUC_{0-t}$ and the extrapolated area from the last measurable concentration to infinity, calculated as:

(1) $$AUC_{0-\infty }=AUC_{0-t}+\frac{C_{last}}{k_{el}}$$

where $C_{last}$ is the final observed concentration and $k_{el}$ is the terminal final elimination constant estimated from the slope of the log-linear portion of the concentration–time curve.

### 5.3. Statistical Analysis

Statistical analysis was performed using RStudio statistical software Version 1.2.5033 ^®^ 2009–2019 RStudio, Inc. (Boston, MA, USA). Four replicates were performed per experiment, and comparisons were made using multiple regression analyses. For the in vivo organ index evaluation, data were analyzed using ANOVA under a factorial arrangement, investigating the effects of both treatment and dose as well as their interaction. When significant differences were observed (*p* < 0.05), means were compared using Tukey’s post hoc test. Differences were considered statistically significant at *p* < 0.05.

## 6. Conclusions

Extracts from the *Sechium* H387 07 hybrid and M16 segregant exhibited small differences in their phytochemical composition. Overall, the extracted yield was higher for *Sechium* H387 07 than for the 387 M16 segregant. HPLC analysis also revealed the presence of phytochemicals such as cucurbitacins in the H387 M16 segregant, making it a promising candidate for future bioassays.

Additionally, antioxidant activity studies showed that the *Sechium* H387 07 hybrid had a higher antioxidant capacity than the 387 M16 segregant, which is consistent with the results of previous studies. In terms of safety, the cardiac index, spleens, livers, kidneys, and brains of mice treated with the *Sechium* H387 07 extract did not show significant alterations, suggesting its potential use in humans at controlled doses.

In this study, we also uncovered the most abundant secondary metabolites in the *Sechium* H387 07 extract, including CuB and CuIIA, the latter being reported for the first time in mouse serum after treatment with the hybrid extract. This finding could contribute to our understanding of the anticancer mechanism of *Sechium* H387 07. Finally, general estimates of the pharmacokinetic intervals of key components of the extract, such as C_max_, T_1/2_, and V_d_, were obtained, with special emphasis on apigenin, phloretin, CuB, and CuE. These showed variations with respect to the values obtained in other studies, which could be valuable data for adjusting doses and administration times for treatment with *Sechium* H387 07, in addition to guiding future studies to improve the bioavailability of other drugs.

While these findings provide novel insights, this study has some limitations. It relied on a single animal model with restricted sampling intervals, and in vivo data for H387 M16 were not available due to material constraints. Future research should address these limitations to better validate and extend the pharmacokinetic relevance of these genotypes.

## Figures and Tables

**Table 1:** Composition of secondary metabolites in methanolic extracts obtained from *Sechium* H387 07 and H387 M16 determined by preliminary qualitative colorimetric assays: phenols, flavonoids, terpenoids, tannins, saponins, and alkaloids.

Group of Metabolites	*Sechium* H387 07	H387 M16
Tannins	+	−
Phenols	+++	+
Flavonoids	+	+
Alkaloids	−	−
Saponins	+	++
Terpenoids	+++	+++

Intense presence (+++), moderate presence (++), weak presence (+), and absence of compound (−).

**Table 2:** Flavonoids identified and quantified by HPLC in methanolic extracts of *Sechium* H387 07 and H387 M16.

	Segregant 387 M16	*Sechium* H387 07
Flavonoids	(mg of Metabolite/g of Extract)	
Myricetin	0.00134	0.00594
Kaempferol	0.00209	0.00217
Isorhamnetin	0.00247	0.00124
Apigenin	0.0035	0.00249
Rutin	0.0668	0.00993
Morin	0.01840	0.01049
Quercetin	0.01365	0.00628
Catechin	0.14132	0.09205
Hesperidin	0.05008	0.03749
Phloridzin	0.03711	0.02945
Naringenin	0.00510	0.00526
Phloretin	0.00465	0.01233
Total	0.34669	0.21513

**Table 3:** Cucurbitacins identified in the methanolic extract of H387 07 and 387 M16 according to HPLC analysis. N/I: Not identified.

	Segregant H387 M16	*Sechium* H387 07
Cucurbitacins	(mg of Metabolite/g of Extract)	
Cucurbitacin D	N/I	N/I
Cucurbitacin I	0.039	3.061
Cucurbitacin B	2.63	4.595
Cucurbitacin IIA	N/I	N/I
Cucurbitacin E	0.0779	0.594
Total	2.747	8.251

## Data Availability

Data are contained within the article and Supplementary Materials.

## Supplementary Materials

The following supporting information can be downloaded at: https://www.mdpi.com/article/10.3390/molecules30193948/s1 Figure S1. Chemical structures of the twelve flavonoids identified in *Sechium* H387 07 and H387 M16 (Table 2): myricetin, kaempferol, isorhamnetin, apigenin, rutin, morin, quercetin, catechin, hesperidin, phloridzin, naringenin, and phloretin. Structures were obtained from PubChem (National Center for Biotechnology Information, 2023). Figure S2. Cardiac, splenic, hepatic, renal, and cerebral indices in mice treated with the extract at different doses (8, 125, and 250 mg/kg) compared to control and vehicle groups, evaluated at different time points (0–48 h). Indices are expressed as the ratio of organ weight to body weight. Values represent mean ± standard deviation (*n* = 4). Figure S3. Urinary excretion profile of hesperidin after oral administration of the Sechium H387 07 extract in mice at doses of 8, 125, and 250 mg/kg. Vehicle-treated animals (PBS) are shown for comparison. Data are expressed as mean ± SEM (*n* = 4 per group). Figure S4. Concentration–time profiles of cucurbitacins in mice after oral administration of the Sechium H387 07 extract at doses of 8, 125, and 250 mg/kg. Vehicle-treated animals (PBS) are shown for comparison. (A) Liver concentration of cucurbitacin D (CuD); (B) liver concentration of cucurbitacin E (CuE); and (C) liver concentration of cucurbitacin I (CuI). Data are expressed as mean ± SEM (*n* = 4 per group). Figure S5. Urinary concentration–time profile of cucurbitacin E (CuE) after oral administration of the Sechium H387 07 extract in mice at doses of 8, 125, and 250 mg/kg. Vehicle-treated animals (PBS) are shown for comparison. Data are expressed as mean ± SEM (*n* = 4 per group). Figure S6. Concentrations of cucurbitacin IIa (CuIIa) in mice after oral administration of the Sechium H387 07 extract at doses of 8, 125, and 250 mg/kg. Vehicle-treated animals (PBS) are shown for comparison. (A) Liver concentrations; and (B) urine concentrations. Data are expressed as mean ± SEM (*n* = 4 per group). Table S1. Pharmacokinetic parameters of secondary metabolites identified in *Sechium* H387 07. Where, C_max_ (maximum concentration), T_max_ (time to reach maximum concentration), AUC (area under the curve), AUC_0−t_ (from zero to the last measurable concentration), and AUC_0 ∞_ (from zero to infinity), T_1/2_ (half-life), V_d_ (volume of distribution). Data are expressed as mean ± SD (*n* = 4).
