# Supplementary material for: Pharmacokinetic Profile of Extracts from the Chayote (Sechium edule) H387 07 Hybrid and Phytochemical Characterization of Its Segregant H387 M16 for Potential Therapeutic Applications
Source: Molecules. 2025 Oct 1;30(19):3948. doi: 10.3390/molecules30193948 (PMC12525650; doi:10.3390/molecules30193948)
Supplement: Supplementary file 1 [file molecules-30-03948-s001.zip › molecules-3788264-supplementary.pdf]

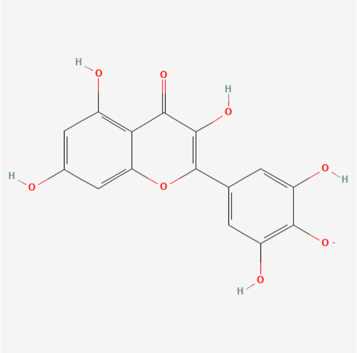	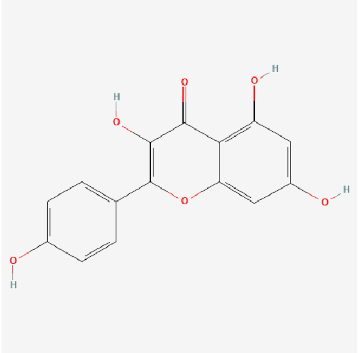	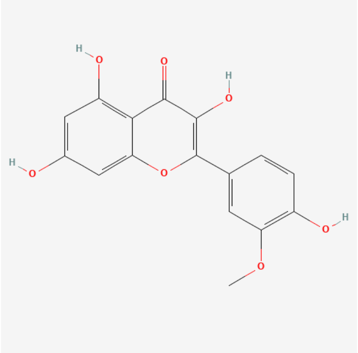
**1.** Myricetin	**2.** Kaemferol	**3.** Isorhamnetin
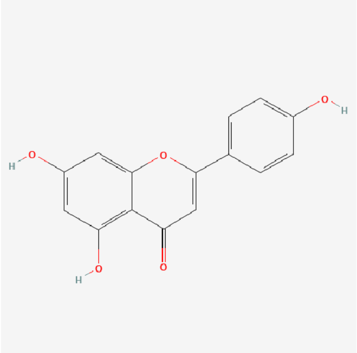	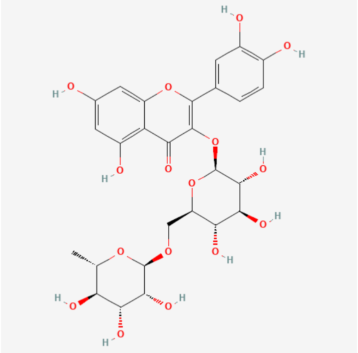	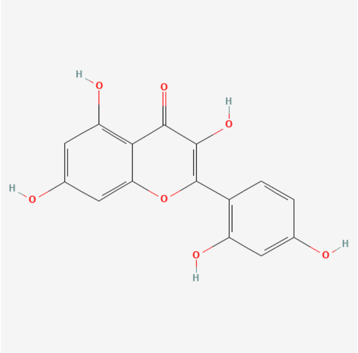
**4.** Apigenin	**5.** Rutin	**6.** Morin
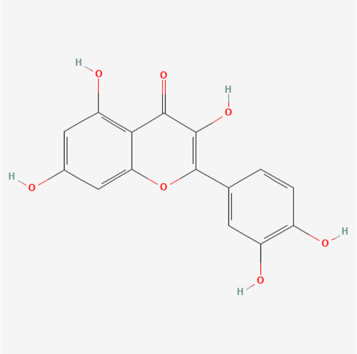	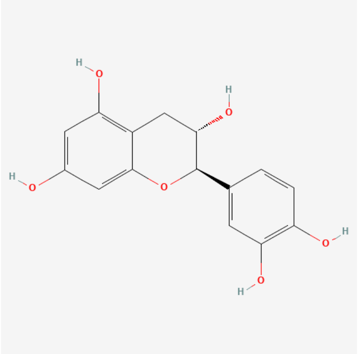	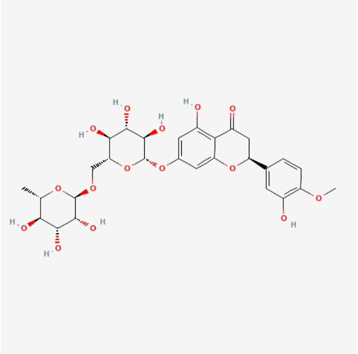
**7.** Quercetin	**8.** Catechin	**9.** Hesperidin
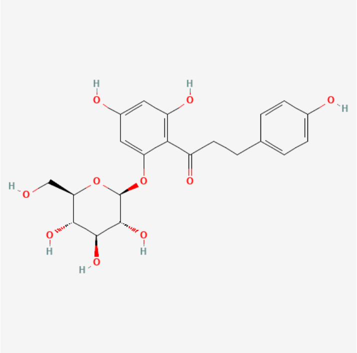	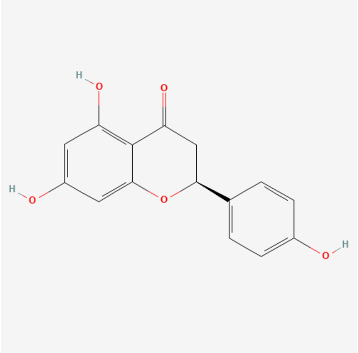	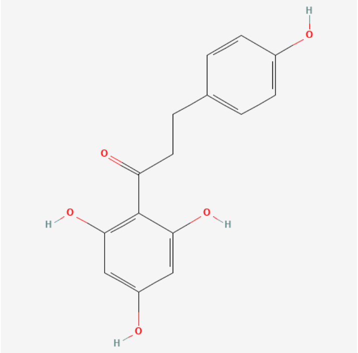
**10.** Phloridzin	**11.** Naringenin	**12.** Phloretin

Supplementary Figure S1. Chemical structures of the twelve flavonoids identified in *Sechium* H387 07 and H387 M16 (Table 2): myricetin, kaempferol, isorhamnetin, apigenin, rutin, morin, quercetin, catechin, hesperidin, phloridzin, naringenin, and phloretin. Structures were obtained from PubChem (National Center for Biotechnology Information, 2023).

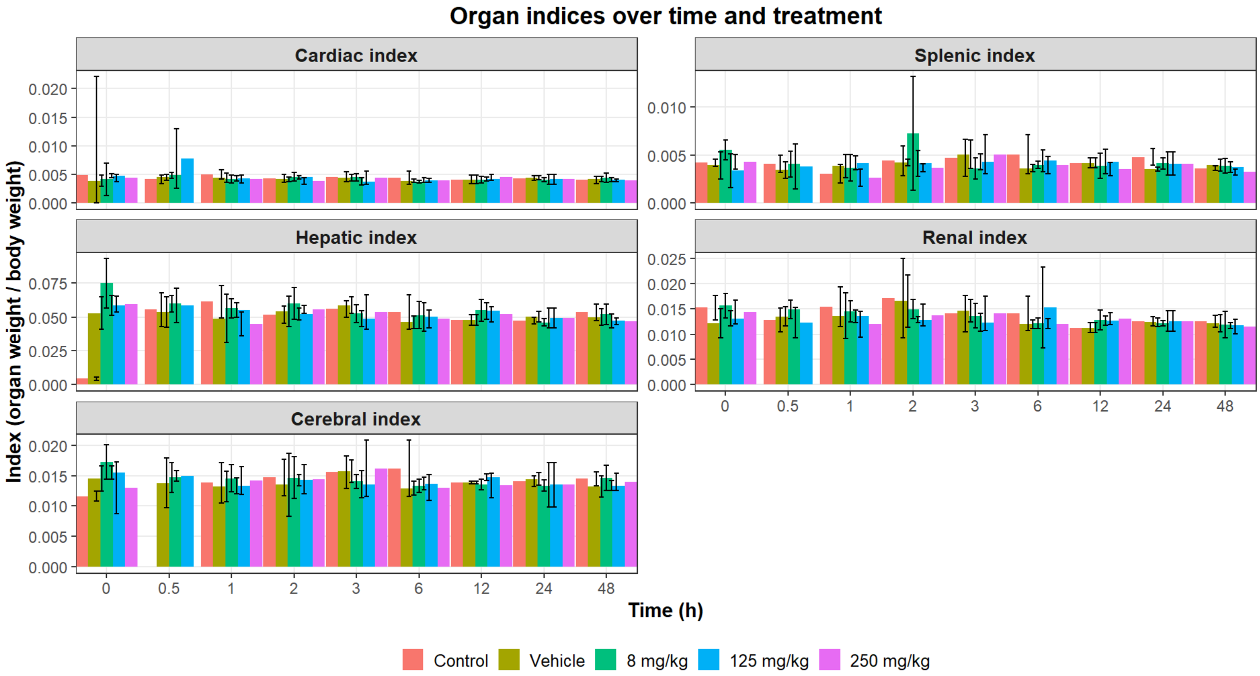

Supplementary Figure S2. Cardiac, splenic, hepatic, renal, and cerebral indices in mice treated with the extract at different doses (8, 125, and 250 mg/kg) compared to control and vehicle groups, evaluated at different time points (0–48 h). Indices are expressed as the ratio of organ weight to body weight. Values represent mean ± standard deviation (n = 4).

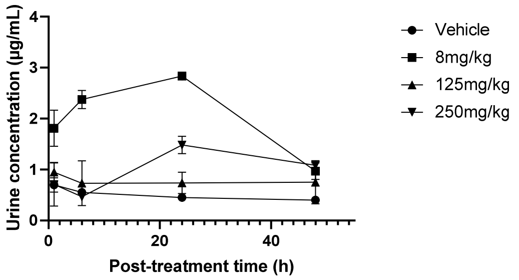

Supplementary Figure S3. Urinary excretion profile of hesperidin after oral administration of the *Sechium* H387 07 extract in mice at doses of 8, 125, and 250 mg/kg. Vehicle-treated animals (PBS) are shown for comparison. Data are expressed as mean ± SEM (n = 4 per group).

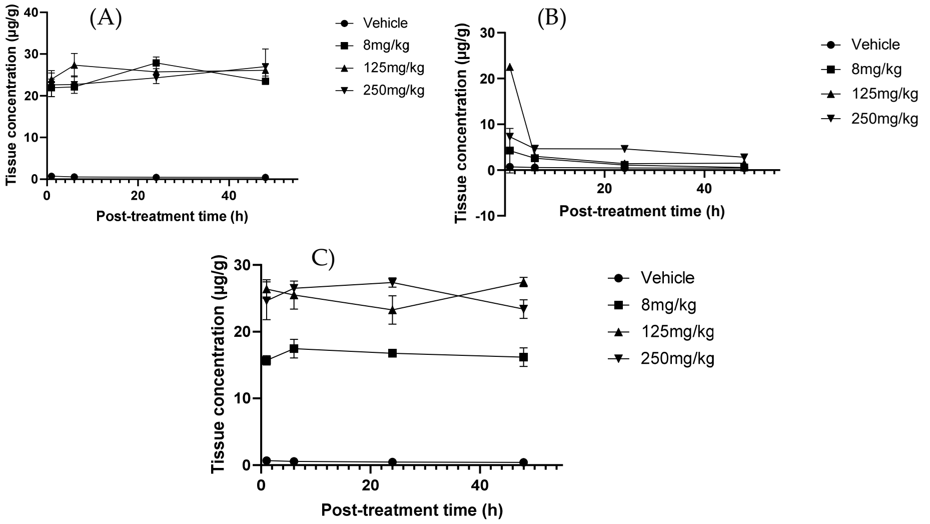

Supplementary Figure S4. Concentration–time profiles of cucurbitacins in mice after oral administration of the *Sechium* H387 07 extract at doses of 8, 125, and 250 mg/kg. Vehicle-treated animals (PBS) are shown for comparison. (A) Liver concentration of cucurbitacin D (CuD); (B) liver concentration of cucurbitacin E (CuE); and (C) liver concentration of cucurbitacin I (CuI). Data are expressed as mean ± SEM (n = 4 per group).

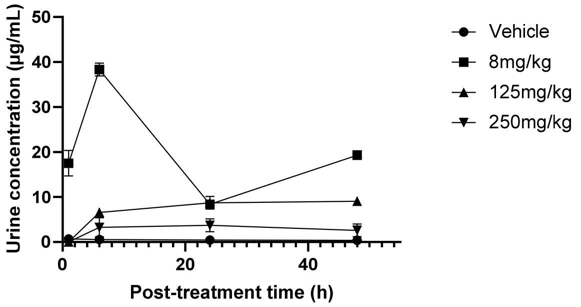

Supplementary Figure S5. Urinary concentration–time profile of cucurbitacin E (CuE) after oral administration of the *Sechium* H387 07 extract in mice at doses of 8, 125, and 250 mg/kg. Vehicle-treated animals (PBS) are shown for comparison. Data are expressed as mean ± SEM (n = 4 per group).

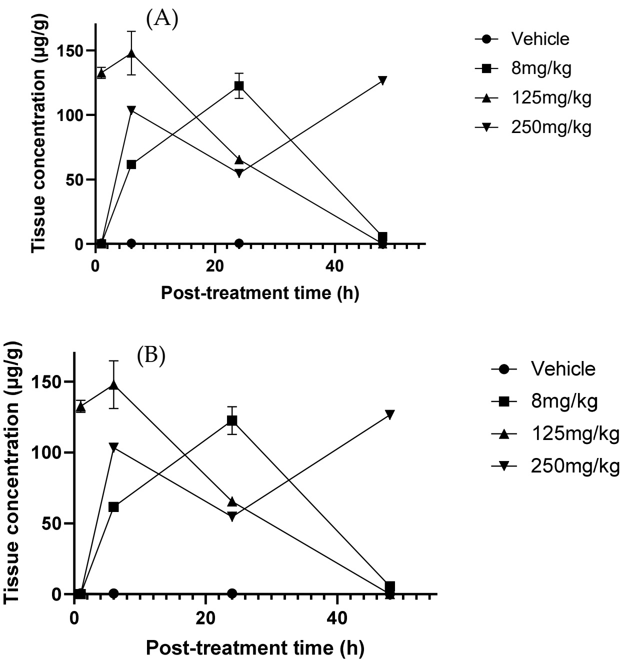

Supplementary Figure S6. Concentrations of cucurbitacin IIa (CuIIa) in mice after oral administration of the *Sechium* H387 07 extract at doses of 8, 125, and 250 mg/kg. Vehicle-treated animals (PBS) are shown for comparison. (A) Liver concentrations; and (B) urine concentrations. Data are expressed as mean ± SEM (*n* = 4 per group).

Supplementary Table S1. Pharmacokinetic parameters of secondary metabolites identified in *Sechium* H387 07. Where, C_{max} (maximum concentration), T_{max} (time to reach maximum concentration), AUC (area under the curve), AUC_{0-t} (from zero to the last measurable concentration), and $AUC_{0\ \infty}$ (from zero to infinity), $T_{1/2}$ (half-life), V_d (volume of distribution). Data are expressed as mean ± SD (*n*=4).

Component	Dose	Pharmacokinetic Parameters
Apigenin	8 mg/kg	C_{max} = 0.85 ± 0.07 µg/mL<br>T_{max}=1.5± 0.3<br>AUC_{0-t}= 2.12 ± 0.15 µg·h/mL<br>$AUC_{0\ \infty}$= 2.39 ± 0.21 µg·h/mL<br>$T_{1/2}$ = 5.6 ± 0.3 h<br>V_d= 3.4 ± 0.5 L/kg<br>Clearance = 0.82 ± 0.09 L/h/kg
Phloretin	8 mg/kg	C_{max} = 1.12 ± 0.09 µg/mL<br>T_{max}=2.0 ± 0.4<br>AUC_{0-t}= 3.45 ± 0.31 µg·h/mL<br>$AUC_{0\ \infty}$= 6.1 ± 0.9 µg·h/mL<br>$T_{1/2}$ = 4.2 ± 0.6 h<br>V_d= 0.95 ± 0.11 L<br>Clearance = 0.82 ± 0.09 L/h/kg
Cucurbitacin B	125 mg/kg	C_{max} = 37.56 ± 0.05 µg/mL<br>T_{max}=1 ± 0.2<br>AUC_{0-t}= 1.87 ± 0.13 µg·h/mL<br>$AUC_{0\ \infty}$= 2.05 ± 0.18 µg·h/mL<br>$T_{1/2}$ = 7.2 ± 1.1 h<br>V_d= 5.0 ± 0.7 L/kg<br>Clearance = 0.70 ± 0.08 L/h/kg
Cucurbitacin E	8 mg/kg	C_{max} = 0.42 ± 0.04 µg/mL<br>T_{max}=2.5 ± 0.3<br>AUC_{0-t}= 1.45 ± 0.11 µg·h/mL<br>$AUC_{0\ \infty}$= 1.80 ± 0.16 µg·h/mL<br>$T_{1/2}$ = 8.4 ± 1.2 h<br>V_d= 4.8 L/kg<br>Clearance = 0.65 ± 0.07 L/h/kg
